# *Staphylococcus aureus* Nasal Carriage and Autoimmune Diseases: From Pathogenic Mechanisms to Disease Susceptibility and Phenotype

**DOI:** 10.3390/ijms20225624

**Published:** 2019-11-11

**Authors:** Fulvia Ceccarelli, Carlo Perricone, Giulio Olivieri, Enrica Cipriano, Francesca Romana Spinelli, Guido Valesini, Fabrizio Conti

**Affiliations:** Lupus Clinic, Rheumatology, Dipartimento di Medicina Interna e Specialità Mediche, Sapienza Università di Roma, 00161 Rome, Italy; carlo.perricone@gmail.com (C.P.); giulio.olivieri19@gmail.com (G.O.); enrica.cipriano@gmail.com (E.C.); francescaromana.spinelli@uniroma1.it (F.R.S.); guido.valesini@uniroma1.it (G.V.); fabrizio.conti@uniroma1.it (F.C.)

**Keywords:** autoimmune diseases, *Staphylococcus aureus*, colonization, pathogenesis, phenotype

## Abstract

The role of infective agents in autoimmune diseases (ADs) development has been historically investigated, but in the last years has been strongly reconsidered due to the interest in the link between the microbiome and ADs. Together with the gut, the skin microbiome is characterized by the presence of several microorganisms, potentially influencing innate and adaptive immune response. *S. aureus* is one of the most important components of the skin microbiome that can colonize anterior nares without clinical manifestations. Data from the literature demonstrates a significantly higher prevalence of nasal colonization in ADs patients in comparison with healthy subjects, suggesting a possible role in terms of disease development and phenotypes. Thus, in the present narrative review we focused on the mechanisms by which *S. aureus* could influence the immune response and on its relationship with ADs, in particular granulomatosis with polyangiitis, rheumatoid arthritis, and systemic lupus erythematosus.

## 1. Introduction

The multifactorial etiology of autoimmune diseases (ADs) has been widely confirmed and in the last years several researches focused on the identification of environmental factors able to induce disease development in genetically susceptible individuals [1,2]. The role of infective agents has been historically investigated, in terms of disease pathogenesis but also as comorbidities related to immunosuppressant treatments [3,4]. This attention determined the identification of microorganisms directly involved in disease pathogenesis. This is the case of *Porphyromonas gingivalis,* characterized by the ability to induce citrullination, a post-translational modification playing a central role in rheumatoid arthritis (RA) development [5,6].

The link with infections has been strongly reconsidered in the last years since the role of the microbiome in AD pathogenesis has been identified [7]. The presence of dysbiosis in patients affected by immune-mediated diseases has been widely demonstrated [8]. In particular, qualitative modifications have been observed in the gut microbiome of different inflammatory diseases in comparison with healthy subjects. Certainly, more evidence is available for spondyloartrhitis, but some studies have confirmed this aspect also in RA and systemic lupus erythematosus (SLE) patients [8,9,10,11]. These modifications have been related to different disease phenotypes and activity degrees [9,10,11].

Despite the great attention focused on gut microbiome modifications, the microbial composition significantly varies across different body sites and microbial communities could be implicated in human health and diseases [12]. The attention to the skin microbiome derives from the evidence that the skin is the largest organ of human body, inhabited by a variety of microorganisms, such as bacteria, fungi and viruses. Many of these microorganisms are harmless or even beneficial to their host, protecting it from invasion by more pathogenic or harmful organisms [13]. Moreover, these different microbial communities could create specific ecological niches, helping in disease prevention or, conversely, contributing to disease development [13]. Exogenous and endogenous factors regulate the growth of particular microorganism families. Among these, we can mention host factors (sex, age), the environment (climate, geographical location), skin topography, immune system (previous exposures to microorganisms, inflammatory conditions) [14].

Interestingly, at a skin level, innate and adaptive immune responses could modulate the resident microbiome, but the microbiome could also influence the immune system [14]. The modification of the skin ecosystem could alter this balance, resulting in different pathogenic conditions. For instance, some bacterial species limit the growth of other bacteria by hydrolyzing sebum lipids to toxic fatty acids [15,16]. Similarly, large-sale alterations of skin microbial communities have been linked to several non-infectious diseases, such as atopic dermatitis, psoriasis, rosacea and acne [14].

*Staphylococcus aureus* (*S. aureus*) is one of the most important component of the skin microbiome, responsible for different infective diseases, with a great range of severity. Epidemiological data suggest for *S. aureus* strains a specific geo-spatial predominance, but clear associations with specific phenotypes have not been reported [17]. Moreover, asymptomatic *S. aureus* carriage could occur in 20–30% of the general population, with a prevalent localization in anterior nares; this prevalence significantly increases in patients affected by ADs [18].

Moving from these evidences, here we performed a narrative review focusing on the possible role of nasal carriage in ADs development and phenotypes. In particular, we aimed at reviewing the impact of *S. aures* colonization on the immune response and AD phenotypes. For this purpose, a literature search was done in PubMed, accessed via the National Library of Medicine PubMed interface (http://www.ncbi.nlm.nih.gov/pubmed). Firstly, PubMed was searched using the term “*Staphylococcus aureus*” in combination with (AND) “autoimmune diseases” OR “autoimmunity”.

Secondly, the same PubMed research was combined with other terms, such as “pathogenesis” OR “immune response” OR “granulomatosis with polyangiitis” OR “rheumatoid arthritis” OR “systemic lupus erythematosus”. Further relevant data were obtained from the reference lists of articles returned using these search terms.

## 2. Staphylococcus aureus: General Aspects

*S. aureus* is the most important species in the *Staphylococcus* genus. It is a Gram-positive, aerobe and facultative anaerobe bacterium, colonizing the human skin. *S. aureus*-related infections are widely spread and can be very serious, with a great variation in mortality, depending on type, virulence factors of involved strains and, finally, patients’ characteristics [19]. Table 1 reports the different factors conferring virulence to this bacterium.

All these factors are relevant from an epidemiological and clinical point of view, contributing to the pathogenicity of *S. aureus* [19]. First of all, several surface structures can play a role by binding extracellular proteins, such as matrix molecules, thus facilitating the host colonization [17]. Moreover, up to 40 exotoxins have been described so far; they are characterized by specific properties, even though a similar structure has been described. They are able to induce T and B cells proliferation and the consequent production of different cytokines. Thus, these exotoxins could modulate the host immune system during *S. aureus* infection [21]. T cell superantigens (SAgs) represent the largest family produced by *S. aureus,* with strong resistance to proteolysis [22]. Moreover, SAg seems to be involved in antigen presenting cells, resulting in polyclonal T cell proliferation, followed by a state of anergy [22].

Moreover, *S. aureus* produces other virulence factors displaying enzymatic properties. We can differentiate cofactors activating host zymogens from enzymes able to degrade tissue components [20]. In particular, exoenzymes—i.e., nucleases and proteases—act by cleaving and inactivating different molecules involved in host defense, such as complement factors, antimicrobial peptides, surface receptors. Nonetheless, other exoenzymes could modify endothelial and epithelial barriers by cell lysis and cleavage of junction proteins [20].

Additionally, the ability of forming biofilm should be considered. In fact, biofilms are crucial for the colonization of medical devices—such as prothesis or catheters—and contributes to *S. aureus* spread into hospital settings, also fostering the resistance to antimicrobial therapy [23].

*S. aureus* is one the most frequent cause of skin infections, but it could be responsible for other serious conditions [19]. We summarize these infections in Table 2.

Given this huge spectrum of infective phenotypes, it is easily understandable how *S. aureus* could be considered one of the most concerning existing pathogen.

## 3. *Staphylococcus aureus* Nasal Carriage: Interplay with the Immune System

Nasal swab cultures demonstrate that 20–30% of the healthy population is persistently colonized by *S. aureus* and 30% are intermittently colonized [62,63]. The different risk among persistent and intermittent carriers was not enough defined. Nonetheless, data from the literature report a lower *S. aureus*-attributable mortality for colonized patients developing bacteremia than non-colonized subjects. This observation could suggest a staphylococcal-specific immune *priming* or immunomodulation occurring as a consequence of colonization, impacting on the outcome of infection [64].

The principal habitat for *S. aureus* is represented by the squamous epithelium in the anterior nares; nonetheless, the posterior nares could be colonized. This selected colonization site is probably related to the presence of several components on bacterium surface—such as clumping factor A and B-, able to directly interact with nasal epithelium, promoting adhesion [65].

**Figure 1:**
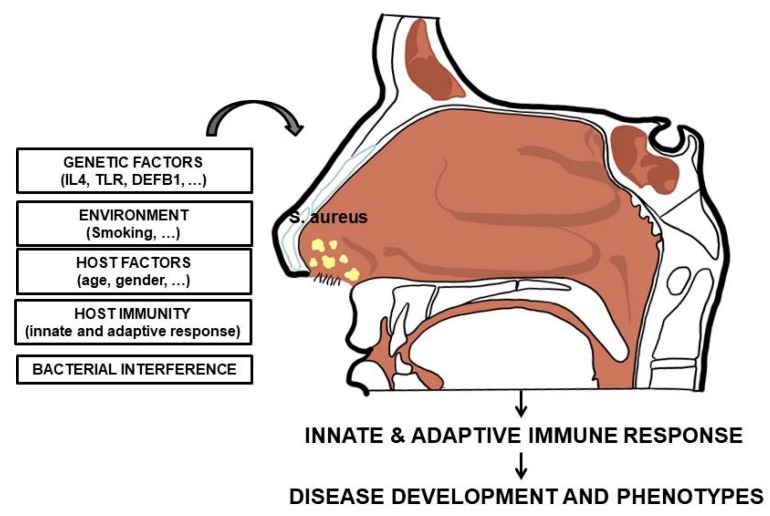
Host and bacterial factors influencing *S. aureus* nasal colonization.

The condition of carriage, potentially associated with an increased infective risk, results from a complex interplay between several host and bacterial factors [66]. The different involved factors are reported in Figure 1.

In particular, several indirect evidences suggested the connection between the host immune system and *S. aureus* nasal carriage. Certainly, a multitude of factors could mediate the equilibrium with the host immune response, but the specific mechanisms underlining this equilibrium is unknown. Specific polymorphisms on genes encoding for mannose-binding lectin, Toll-like receptor 2 (TL2), glucocorticoid receptor, C-reactive protein, β-defensin 1 promoter region and interleukin-4 have been associated with this condition [67,68,69,70,71,72,73].

These associations underline the involvement of both innate and adaptive immunity during *S. aureus* colonization. Moreover, disease characterized by immune system depletion shows an increased incidence of colonization. For instance, this has been demonstrated in HIV patients, in which a CD4+ T cell depletion is observed, but also in patients affected by ADs treated by immunosuppressant drugs [74,75].

Moving on to innate immunity, the epithelium barrier is the first line defense in the nose. In particular, keratinocytes could express several antimicrobial peptides, including LL-37 and β-defensin-3, as well as chemokines (i.e., IL8) and cytokines (IL6, TNF, IL10) [76,77,78].

Among antimicrobial peptides, human β-defensin-3 is the most important peptide in terms of in vitro anti-bacterial effects and involvement in skin infection development. It is produced by the nasal epithelium in response to the presence of *S. aureus* or to skin barrier alterations, suggesting a role in bacterium clearance in colonized and infective patients. Nonetheless, persistent carriers subjects show reduced level of this antimicrobial peptide in comparison with non-carriers [76,78,79,80].

Furthermore, LL-37 shows a strong in vitro killing effect on *S. aureus*: however, similar to that observed for human β-defensin-3, higher level of LL-37 have been observed in colonized patients [81,82].

At this level, TL2 plays a crucial role for keratinocyte-mediated *S. aureus* recognition, leading to immune response activation, with T cell recruitment [80]. In details, as widely demonstrated, TLR2 acts by recruiting intracellular TIRAP, MyD88, IRAK-1 and 4, leading to the activation of two different pro-inflammatory pathways—specifically, NF-κB and MAPKs. These processes translate into the production of pro-inflammatory cytokines and chemokines, crucial to recruit effector T cells, in particular Th1 and Th17 [83]. Interestingly, Parker and colleagues in 2012 suggested the possible role of TLR9 as a critical receptor mediating the induction of type I IFN signaling in dendritic cells in response to *S. aureus* colonization [84]. These results, confirmed by Parcina et al. in 2013, demonstrated an additional mechanism by which *S. aureus* could influence the innate immune response to infections [85]. However, neutrophils represent the first line cells intervening in *S. aureus* clearance: the bacterium is eliminated by phagocytosis and destroyed by hypochloric acid and oxygen radicals. Furthermore, activated neutrophils could release DNA into the extracellular matrix to produce neutrophil extra traps (NETs), enhancing the cytotoxicity of antimicrobial agents. Following the uptake of bacteria, neutrophils typically undergo apoptosis and are cleared by macrophages, resulting in microbe clearance [86,87].

More recently, several pieces of evidence have suggested that neutrophils and macrophages are in fact not able to kill all bacteria, and that *S. aureus* could proliferate at an intracellular level. In particular, a reduced number of bacteria were not removed from blood and remained localized in the neutrophils and could play a role in bacterial dissemination. Studies conducted on murine models and humans demonstrated the ability of *S. aureus* to survive in neutrophils, suggesting the so-called “Trojan Horse” theory of dissemination [88]. Moreover, *S. aureus* has been demonstrated able to survive in other cells, in particular macrophages and endothelial cells [89,90].

**Figure 2:**
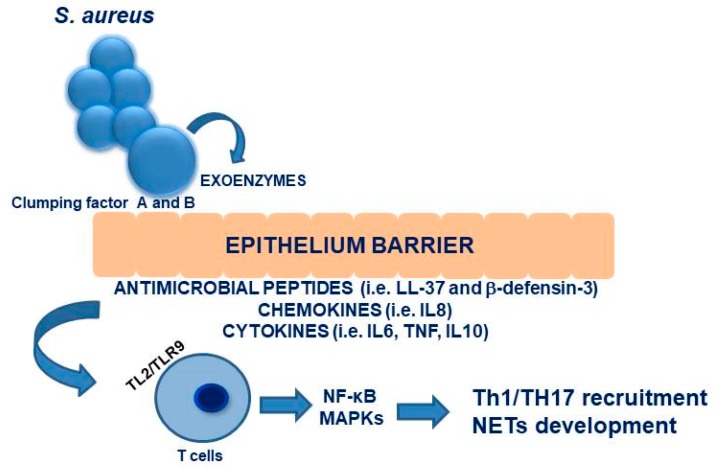
Schematic representation of interactions between nasal epithelium and immune response.

The dissemination occurs in two phases: in the first one, *S. aureus* bacteria replicate and escape from the macrophages by lysis during the first 24 h of infection. In the second phase, bacterial levels increase in organs other than the liver, such as the kidneys [91]. Figure 2 summarizes the interactions between the nasal epithelium and immune responses.

As recently demonstrated, pathogens could activate specific transcriptional pathways interfering with host defense mechanism. Several efforts have been made to identify the role of these new pathways in *S. aureus* infections. Among these, the pathway involving activation of Stimulator of IFN Genes (STING) has been identified. STING play a key role in the host immune response, by exerting the recognition of nucleotide and allowing the identification of different microorganisms [92].

In particular, STING is able to recognize cyclic dinucleotides or, in association with intracellular sensors, DNA from bacteria, viruses and host cells, in order to control the host immune responses [93]. Several lines of evidence have demonstrated STING functions during viral infections; conversely few data are available about its possible role in bacterial pathogenesis [94]. Nonetheless, the STING activation, leading to IFNβ production, may be beneficial or detrimental for the host according to the specific infection. The role of STING depends on the influence of cyclic dinucleotides in bacterial metabolism, such as biofilm formation and protein function [95]. Concerning the possible role of STING pathways in *S. aureus* infection, Sciumpia and colleagues suggested that TLR and STING pathways could play opposite roles in host defense against this bacterium, by using a cutaneous infection model. In fact, the lack of TLR signaling leads to a reduced production of IL-1β and neutrophil recruitment. In contrast, the absence of STING signaling enhances the ability to restrict the infection. Thus, the authors hypothesized that opposing roles of TLR and STING could play a key role in the complex interplay of innate immune signaling pathways triggered by *S. aureus* [96].

Contrary to these data about innate immunity, the development of an adaptive immunological response to *S. aureus* is not fully understood and still remains unclear. However, some studies have observed a different humoral immune response by comparing carrier and non-carrier *S. aureus* subjects. In particular, carrier subjects shown higher serum IgG titres, higher median levels of IgA for several staphylococcal proteins, and higher titres of neutralising antibodies to superantigens specifically expressed by their resident strain [97,98,99]. However, the mechanism by which *S. aureus* colonization could induce a humoral response has not been clarified. This specific aspect of immunological response to *S. aureus* is very intriguing in the light of possible development of vaccines against this bacterium.

## 4. *Staphylococcus aureus* and Granulomatosis with Polyangiitis

Granulomatosis with polyangiitis (GPA) is a small-vessel vasculitis characterised by granulomatous inflammation, involving hebupper respiratory tract (URT) and lower respiratory tract. The URT involvement determined the interest on the possible role of nose microbiome on disease pathogenesis, with particular attention on *S. aureus* carriage. Up to 80% of GPA patients shows the involvement of nasal mucosa, often as first involved site before the development of a systemic autoimmune vasculitis, characterized by the positivity for specific anti-neutrophil cytoplasmic autoantibodies directed against proteinase 3 [100].

From an epidemiological point of view, a nasal *S. aureus* carriage condition is significantly more frequent in GPA patients than healthy individuals and patients with chronic rhinosinusitis [101]. In particular, 60–70% of GPA patients are chronic nasal carrier for *S. aureus*, compared with 20–30% of healthy subjects [102,103,104]. Moreover, this bacterium seems to play a role in determining disease relapses [105]. Stegeman and collegues in 1994 analysed 71 biopsy-proven GPA subjects, showing that *S. aureus* nasal carrier patients were more prone to disease relapse. In particular, the risk of disease relapses has been described seven times higher in chronic nasal carriers [53]. This result was confirmed by Zycinska and colleagues in 2008, showing a 4.5-fold risk for relapse in limited GPA [103].

Finally, this association has not been definitively confirmed, as demonstrated by the study published by Laudien et al., showing only a trend for a higher relapse rate [100], in agreement with previous data deriving from a Norwegian cohort [105]. More recently, a multicentre prospective study conducted by Salmela and colleagues, including 73 newly diagnosed GPA, demonstrated a significantly higher relapse rates in chronic *S. aureus* carriers [106].

To support the association between *S. aureus* colonization and disease flare-ups, two randomized clinical trials demonstrated the ability of treatment with trimethoprim-sulfamethoxazole in preventing relapses [106,107]. The beneficial effect of antibiotic treatment has been demonstrated especially in GPA patients with localized disease but not in those with a systemic involvement [105,106].

The study recently published by Rhee and colleagues aimed at examining the entire community of nasal bacteria and fungi in 60 GPA patients and 41 healthy controls. *S. aureus*, *S. epidermidis*, *Corynebacterium tuberculostearicum*, and *Propionibacterium acnes*, resulted the most prominent bacterial species in the GPA nasal cavities. The authors identified a significant difference in weighted UniFrac between GPA participants and healthy controls, suggesting a different microbial composition between the two groups [108]. More recently, Lamprecht and colleagues evaluated the samples from nasal cavities by 29 GPA, 21 RA patients, and 27 healthy controls; the authors performed a 16S rRNA amplicon sequencing comparing nasal microbiome in patients and controls, showing a trend for a reduced diversity and a decrease in taxonomic abundance and microbial richness in the samples from GPA compared with RA patients. Finally, alpha diversity—defined as mean species diversity in sites or habitats—resulted reduced in GPA patients with active disease in comparison with patients showing a remission status. GPA and RA patients displayed similarities at the family levels, in particular increased abundance of *Planococcaceae,* but also some differences in microbial composition. In fact, GPA patients showed abundance of *Prevotellaceae* in comparison with RA. The authors concluded that both immunosuppressive status and disease background could influence the URT microbiome composition. The analysis of GPA patients revealed that bacterial composition at the phylum, class and family level was not different according disease activity status, except for the *Staphylococcaceae* family, resulting more abundant in remission condition. The application of qPCR demonstrated a significant higher proportion of *S. aureus* in GPA patients than in RA and healthy controls [109].

Taken together, these data suggested an unbalanced composition of the nasal microbial flora, that could contribute as environmental factor by interacting with a susceptible genetic background, determining disease development and phenotype. Thus, moving on to a disease-related pathogenic scenario, GPA-related granulomatous lesions are characterized by the presence of ectopic lymphoid structures, suggesting the development of an antigen-driven inflammatory response at this level [110,111]. Moreover, the barrier dysfunction and the modification of mucociliary clearance identified in GPA subjects could be associated with the occurrence of dysbiosis [109]. Several studies have investigated the possible mechanisms by which *S. aureus* carriage could play a role in disease development and phenotypes. First of all, its role on the dysbiosis has been suggested, involving the reduced secretion of human β-defensin-3, an epithelial defensin displaying a high activity against *S. aureus* [112].

Moreover, the role of *Staphylococcus* superantigens (SAg) has been investigated. This term refers to a group of antigens that are able to induce global changes in the lymphocyte repertoire, by stimulating more than 5% of the naive lymphocyte pool. In particular, these exotoxins shown a strong ability to stimulate T cells in a non-antigen way. To date, three classes of SAg have been described—staphylococcal enterotoxins, exfoliative toxins and toxic-shock syndrome toxin 1 (TSST-1) [113,114,115].

Popa and colleagues observed an increased risk to relapse in *S. aureus* carriers producing the superantigen TSST-1 [102]. Conversely, Fijolek and colleagues identified the presence of at least one SAg in 34.8% of GPA patients, but its presence was not associated with disease activity. However, earlier studies had not revealed a correlation between the presence of SAg genes and the expansion of specific T cell subsets in peripheral blood [116].

The role of *S. aureus* as a trigger in GPA development has been reconsidered in the last decade, underlining that it could be difficult to distinguish between causality and consequence of *S. aureus* carriage in determining GPA pathological setting. In 2011, Laudien and colleagues evaluated the molecular signatures at the nasal barrier level of a cohort of GPA patients, compared with healthy controls. The authors identified specific GPA-associated molecular profiles; in particular, ten transcripts differentially expressed between case and controls. These transcripts include antimicrobial peptides (human-defensin, lysozyme, DEFB4, S100A7), innate immune receptors (TLR4, NOD-like receptor, C3, CD36), and cytokines (IFNγ, TGFβ1, IL17D). Interestingly, these transcriptional profiles resulted independent from *S. aureus* colonization, as demonstrated by the reduction of the number of significantly dysregulated transcripts in carriers. The authors speculated about a secondary role of *S. aureus* colonization, representing a consequence of initially disturbed nasal barrier function rather than a causative event starting pathological cascade. Moreover, impaired lysozyme expression in GPA patients could promote *S. aureus* colonization; thus, the upregulation of β-defensin family members, might represent a direct antibacterial response and an attempt to restore immunological barrier function [117].

More recently, Wohlers and colleagues aimed to evaluate the ability of *S. aureus* to influence the nasal microenvironment’s cytokine secretion. The authors identified an altered baseline cytokine pattern in GPA subjects, with up-regulation of G-CSF and reduction in IL-8 concentrations. Both nasal epithelial cells from GPA and controls responded to *S. aureus* stimulation, but a significantly lower IL-8 secretion and a reduced dynamic range of response to the stimulus was observed in GPA patients. Therefore, the study suggested that the dysregulation of baseline expression of G-CSF and IL-8 and the reduced response to microbial stimulation could modify the composition of the nasal microbiome and favour an imbalanced inflammatory response, potentially relevant for the disease course [118].

Finally, Glasner and colleagues in 2015 analyzed serum antibody levels against a comprehensive antigens set of *S. aureus,* in order to evaluate humoral immune response against this bacterium. The authors observed that GPA sera contained lower anti-staphylococcal IgG levels than controls, regardless of the treatment assumed. These results could suggest that GPA patients could be less capable of mounting a potentially protective antibody response to *S. aureus* than healthy individuals. Furthermore, the evaluation of 210 *S. aureus* isolates from GPA patients demonstrated a higher diversity, mirroring the general *S. aureus* population [69]. The same authors investigated the gene repertoire of *S. aureus* nasal isolates from PR3-ANCA-positive patients, by performing a comparison between *S. aureus* isolates from MPO-ANCA-positive patients and from healthy controls. The MPO-ANCA-associated *S. aureus* isolates resulted different from healthy controls and PR3-ANCA-associated isolates. Furthermore, several genetic loci of *S. aureus* are associated with either PR3-ANCA- or MPO-ANCA-positive ANCA associated vasculitis, indicating a possible role for pore-forming toxins in PR3-ANCA-positive GPA [119].

## 5. *Staphylococcus aureus* and Other Autoimmune Diseases

Behind the GPA, the possible role of *S. aureus* carriage condition has been investigated in other ADs, in particular RA and SLE, even though without reaching conclusive results. As widely observed, RA patients are five-times more likely to die for pneumonia, have a 10–15 times greater risk for bone and joint infections and are three times more likely to suffer skin infections than the general population [120]. This risk seems to be higher in patients treated with biological drugs, in particular TNF antagonists. This could be related to the role of TNF in the innate immune response to both intra and extracellular infections, including *S. aureus* [121,122,123,124].

Few studies—with limited sample size—have evaluated the prevalence of *S. aureus* nasal colonization in RA cohorts, describing a prevalence of 50–56%, similar to that identified in control subjects [32,125]. Varley et al. in 2013 evaluated a large cohort of patients affected by different immune-mediated inflammatory diseases—RA 28.1%; psoriasis/psoriatic arthritis 52%; ankylosing spondylitis 6.6%; other diseases 11.1%. More than half of enrolled patients (52.0%) were taking biologic therapy at the first assessment (92.6% of them were on TNF inhibitor treatment). At the enrolment, 40.0% of patients were *S. aureus* carriers, with 12.3% of patients showing methicillin-resistant *S. aureus*. The colonization prevalence in anti-TNF treated patients was similar than that observed in non-treated patients; however, this prevalence is significantly higher in psoriasis subjects than RA. A follow-up evaluation was performed in 70% of patients after a median interval of 0.63 years: subjects colonized at baseline were more likely to be colonized also at the follow-up, especially if treated with anti-TNF during this period (OR 2.2) [125]. More recently, Goodman and colleagues observed that RA patients treated by biological drugs showed a statistically significant increase in *S. aureus* colonization (37%) compared to RA on DMARDs alone (24%), or control subjects affected by osteoarthritis (20%). This result was confirmed at logistic regression analysis, after correction for glucocorticoids and antibiotic treatment, recent hospitalization, and diabetes [126]. In RA cohort, 28% of patients was treated by adalimumab, 25.2% by etanercept, 12.2% by abatacept, and 12.2% by rituximab. However, no data about SA colonization rate in the different biological treatments were provided in the study [126].

From a pathogenic point of view, data about the role of *S. aureus* in RA development are lacking. However, Ataee and colleagues in 2014 performed a study to detect staphylococcal enterotoxin C in synovial fluid of 50 RA patients. In the evaluated cohort, bacterial culture was negative in all samples, but interestingly, 66% of analysed samples contained staphylococcal enterotoxin C gene. Moreover, enterotoxin C was detected in 46% of synovial fluid [127]. The same authors also evaluated the enterotoxin D levels in 120 blood and synovial fluid samples from RA patients: in particular the authors performed bacterial DNA extraction from blood buffy coat and from synovial fluid. The study demonstrated entD gene in 50% of patient’s synovial fluid and 37.5% of blood buffy coat. Moreover, the ELISA test demonstrated staphylococcal enterotoxin D in 36.16% of analyzed synovial fluid and 33.33% of blood buffy coat [128]. These results could suggest a possible role of *S. aureus* in RA development, but certainly other studies are needed to confirm this suggestion.

Moving on to SLE, a chronic autoimmune disease potentially involving any organ/system, infections resulted highly associated with the disease onset but also with disease flare-ups [129]. Several studies have suggested the possible pathogenic role of different infective agents, in particular *Epstein-Barr* virus, parvovirus B19, retrovirus, and cytomegalovirus [130,131]. In fact, the prevalence of these infections has been proved significantly higher in SLE cohorts in comparison with healthy subjects. The abovementioned viruses could activate a self-directed immune response, influencing disease development and course [132,133].

Few data are available concerning the possible role of bacteria in SLE pathogenesis. Concerning *S. aureus*, in 2012 Chowdhary and colleagues performed a study on HLA-DQ8 transgenic mice subcutaneously implanted with mini-osmotic pumps, capable of continuously delivering the Sag or staphylococcal enterotoxin B. Chronic exposure to the latter resulted in a multisystem autoimmune inflammatory disease similar to SLE, as demonstrated by mononuclear cell infiltration of lungs, liver, and kidneys, associated with the production of anti-nuclear antibodies and deposition of immune complexes at renal level. The identified inflammatory infiltrates predominantly included of CD4+ T cells. Thus, this study suggests the possible etiological role of chronic exposure to antigen bacterial [133].

To the best of our knowledge only one paper, published by our research group, has focused on *S. aureus* nasal colonization in SLE patients. In this study, we enrolled 84 SLE patients and 154 healthy subjects, showing a similar colonization prevalence in case and controls (21.4% versus 28.6%). By dividing patients according to the presence of nasal *S. aureus*, we identified a specific clinical and serological profile in positive patients, characterized by presence of several autoantibodies (anti-dsDNA, anti-Sm, anti-SSA, anti-SSB, anti-RNP) and renal and skin manifestations [134]. It could be hypothesized that *S. aureus* carriage status, by stimulating the type-I IFN pathway, could induce the production of autoantibodies and therefore the development of the previously mentioned clinical manifestations [135]. Indeed, pathogen-recognizing dendritic cells could activate T cells and take up *S. aureus* through an endocytic mechanism, resulting in the activation of TLR9 signaling [84].

## 6. Conclusions

In the present review, we have focused on the possible role of *S. aureus* nasal colonization on AD development and phenotype. Behind the development of infective diseases with different degrees of severity, *S. aureus* colonization seems to be able to modulate the immune system both in terms of its innate and adaptive responses. Based on these premises, literature data underlines the role of this bacterium firstly in patients affected by GPA from a pathogenic and disease phenotype point of view. Moreover, few data suggest the possible role also in other ADs, such as RA and SLE. Other studies, focusing on these patients, are needed in order to confirm these preliminary suggestions. In conclusion, data from the literature suggest that the assessment of a state of colonization must be taken into consideration especially in specific populations, such as patients affected by ADs treated by immunosuppressant treatments. Further studies are needed in order to evaluate the modifications determined by *S. aureus* colonization in ADs and the impact of bacterium eradication in disease course.

## Figures and Tables

**Table 1:** Factors conferring virulence to *S. aureus*.

Resource	
**Surface Structures [17]**	Polysaccharide capsule Adhesins Protein A
**Exotoxins [20,21]**	Hemolysins (α, β, γ and δ) Panton-Valentine leucocidin (PVL) Epidermolytic toxin A, cromosomal, and B, plasmidic Toxic shock syndrome toxin-1 (TSST-1) Enterotoxins
**Exoenzymes [20]**	Lipases Coagulases Nucleases Staphylochinases Ureases Hyaluronate-lyases Serine-proteases Catalasys Superoxide dismutases

**Table 2:** *S. aureus* related infections.

Infections	Details
**Skin and Soft Tissues Infections [24,25,26,27,28]**	From benign to life threating conditions (impetigo, cellulitis, surgical sites infections, cutaneous abscesses, purulent cellulitis)
**Osteoarticular Infections [29,30,31,32,33,34]**	Osteomyelitis, septic arthritis, prosthetic joint infections
**Pleuropulmonary Infections [35,36,37,38]**	Predominant role in hospital-acquired pneumonias in comparison to community-acquired ones.
**Bacteremia [39,40,41]**	Direct evolution of a local infection
**Meningitis [42,43,44]**	Complication of a primary non-central nervous system infection
**Epidural Abscesses [45,46]**	Rare intracranial or spinal condition, recognized in USA as second most common infection due to malpractice
**Toxic shock Syndrome [47,48,49]**	Sustained by super antigen-mediated process, linked to toxic shock syndrome toxin-1, able to activate T-cells, with massive cytokine release
**Infective Endocarditis [50,51,52]**	Observed in a proportion of *S. aureus* infected patients ranging from 16% to 34%
**Cardiac Devices Infections [53,54]**	Directly occurring during implantation or indirectly via *haematogenous* seeding from a distant source
**Intravascular Catheter Infections [55,56,57]**	Potentially leading to a bacterial spreading in the bloodstream, configuring the so-called central line-associated blood stream infection (mortality rate 7–21%)
**Urinary Tract Infections [58,59]**	Frequent in case of indwelling urinary catheter
**Septic Thrombophlebitis [60,61]**	Reported in up to 8% of all patients with bacteriema

## Abbreviations

Autoimmune diseases	ADs
Rheumatoid arthritis	RA
Systemic lupus erythematosus	SLE
*Staphylococcus aureus*	*S. aureus*
Toll-like receptor 2	TL2
Stimulator of IFN Genes	STING
Granulomatosis with polyangiitis	GPA
Upper respiratory tract	URT
